# Case Report: Chemoimmunotherapy in microsatellite-instability-high advanced goblet cell carcinoma of the colon

**DOI:** 10.3389/fimmu.2023.1160586

**Published:** 2023-07-07

**Authors:** Arda Ulaş Mutlu, Erman Aytaç, Mehmet Gülmez, Sibel Erdamar, Leyla Özer

**Affiliations:** ^1^ Faculty of Medicine, Acibadem University, Istanbul, Türkiye; ^2^ Department of General Surgery, Faculty of Medicine, Acıbadem University, Istanbul, Türkiye; ^3^ Department of Surgery, Grossman School of Medicine, New York University, New York City, United States; ^4^ Department of Pathology, Faculty of Medicine, Acıbadem University, Istanbul, Türkiye; ^5^ Department of Medical Oncology, Faculty of Medicine, Acıbadem University, Istanbul, Türkiye

**Keywords:** chemoimmunotherapy, metastatic colon cancer (mCRC), complete response, MSI-H, microsatellite unstable (high), goblet cell

## Abstract

**Background:**

Mismatch repair (MMR) deficiency is a fundamental factor affecting the management treatment outcomes of colorectal cancer (CRC). MMR status can be diagnosed by both immunohistochemistry (IHC) polymerase chain reaction (PCR). Since tumors with MMR deficiency are prone to respond to immunotherapy immune checkpoint inhibitors are used to treat such tumors.

**Case presentation:**

A 69-year-old male patient presented to an outside clinic with weight loss and abdominal pain. Radiological investigations detected a mesenteric mass of 10 cm, peritoneal implants, and mediastinal lymphadenopathy. The eventual biopsy result from the mesenteric mass was mucinous adenocarcinoma with a goblet cell pattern. Since the IHC result was unclear for deficiency in mismatch repair (dMMR) metastatic CRC (mCRC), the diagnosis was confirmed with PCR. The patient received 8 cycles of FOLFIRINOX + bevacizumab followed by FOLFOX combined with pembrolizumab. No adverse effect was reported related to immunotherapy which resulted in radiologic and metabolic regression. The patient underwent cytoreductive surgery and hyperthermic intraperitoneal chemotherapy (HIPEC). The final pathology results revealed a pathological complete response and R0 resection. In the 6^th^ month follow-up, no recurrence or metastasis was reported.

**Conclusion:**

Chemotherapy and immunotherapy combination is a promising treatment modality which can also be used for mCRC. This is the index case who received chemotherapy in combination with immunotherapy for mucinous adenocarcinoma of the colon with a goblet cell pattern and had pCR.

## Introduction

Colorectal cancer (CRC) with a deficiency in mismatch repair (dMMR) is characterized by a strong mutator phenotype known as high microsatellite instability (MSI-H) and tumor mutation burden (1). The vast majority of MSI-H/dMMR colon cancers exhibit distinctive features, such as the tendency to arise in the proximal colon and comprise a poorly differentiated, mucinous, or signet ring cell component (2). Almost 15% of all CRCs harbor MSI-H/dMMR phenotype.

Microsatellite instable (MSI) disease is characterized by an obvious antitumor immune response, with increased lymphocyte infiltrate that entails the basis for the improved prognosis of especially stage II CRC with MSI. An explanation for the lack of benefit of fluoropyrimidine (FP)-based chemotherapy in these patients is the antagonization of antitumor response by the immunosuppressive effects of chemotherapy (3). Fortunately, MSI has been established as a strong predictor of efficacy in blocking the immune checkpoint, leading to the approval of programmed death 1 inhibitors such as nivolumab ± ipilimumab and pembrolizumab for MSI patients with metastatic CRC (mCRC) (4, 5).

There is no data about the utility of the combination of chemotherapy and immunotherapy in mCRC patients, although it has been the standard of care for many types of advanced cancers, such as head and neck, gastric, cervical, and non-small cell lung cancer (6, 7). We represent a case of MSI-H mCRC whose indeterminate MMR findings were confirmed by PCR and who was treated successfully with chemoimmunotherapy.

## Case

**Figure 1:**
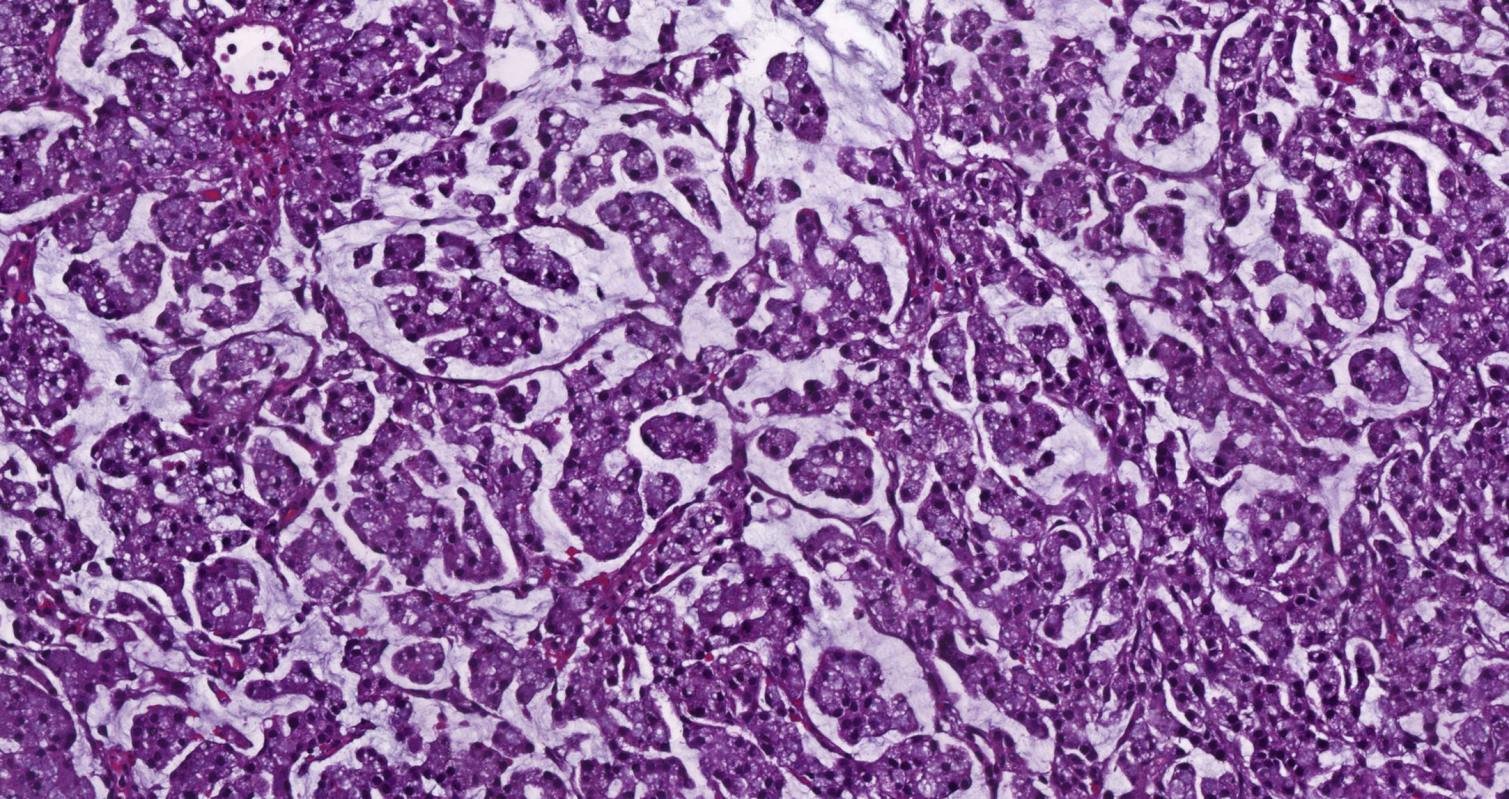
Mucinous adenocarcinoma with a goblet cell pattern, H&E x22.3.

**Figure 2:**
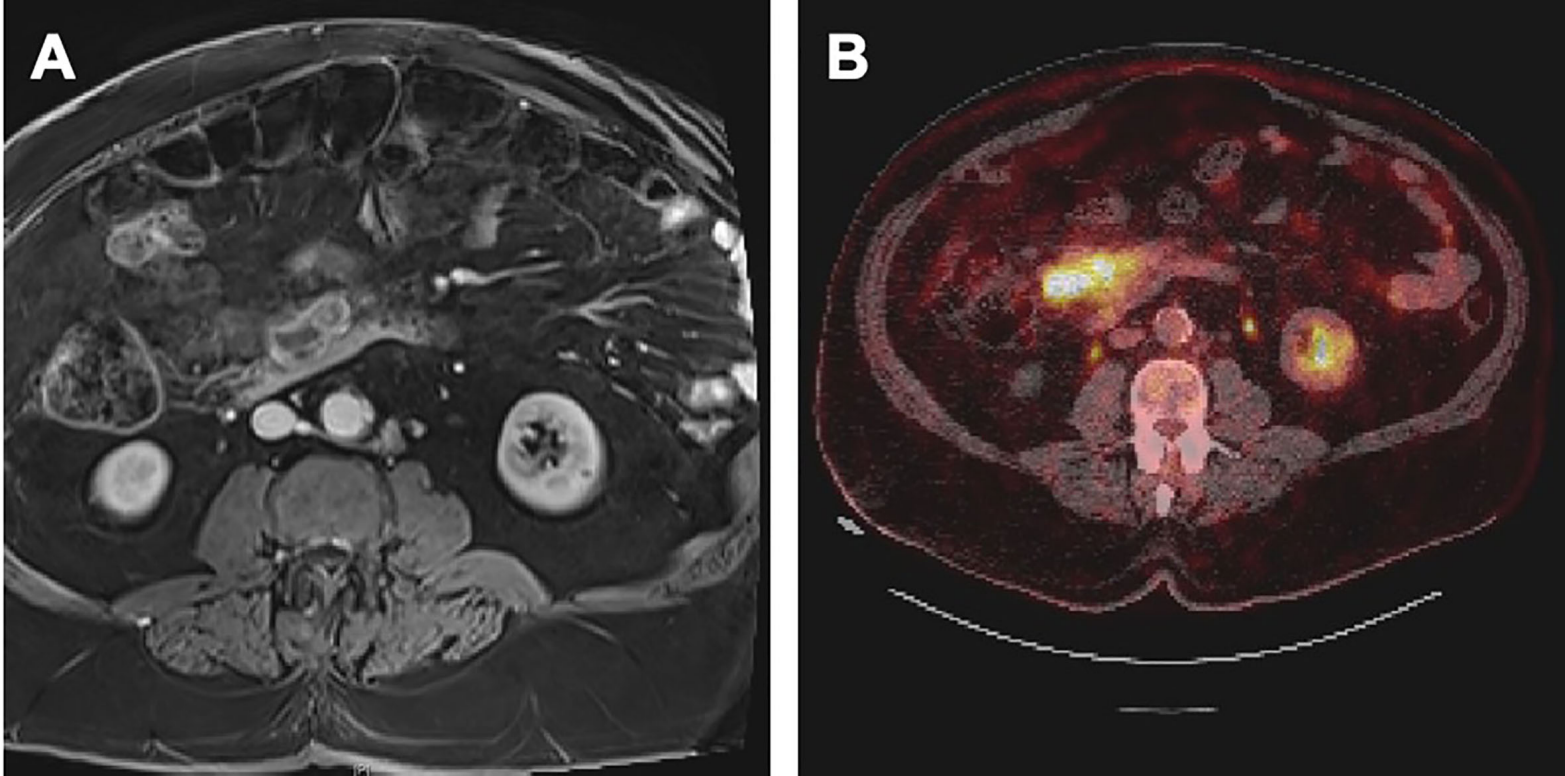
**(A)** MRI at the diagnosis. **(B)** PET/CT at the diagnosis.

A 69-year-old male patient presented to an outside clinic with weight loss and abdominal pain. Radiological investigations detected a mesenteric mass of 10 cm, which was supposed to be originating from the right colon. Diagnostic laparoscopy revealed a retroperitoneal mass with peritoneal implants. Biopsies’ results were carcinoma and its peritoneal metastasis. Then, the patient was referred to our center. A colonoscopy was performed, revealing a 3 cm diameter mass at the base of the cecum. Mucinous adenocarcinoma with a goblet cell pattern and widespread extracellular mucin secretion was detected (**Figure 1**). Abdomen magnetic resonance imaging (MRI) showed a 14x5.5x5 cm conglomerate mass and implants extending to the root of the superior mesenteric vein and attaching to the duodenum with minimal ascites in the pelvis (**Figure 2A**). Thorax computed tomography (CT) pointed at 15x10 mm lymphadenopathy in the mediastinum with no other abnormal finding. Molecular analysis of the primary tumor revealed BRAF V600E mutant, KRAS, NRAS wild, and HER-2 (-) disease. Positron emission tomography/computed tomography (PET/CT) scan revealed conglomerated lesion in the mesentery (SUVMax 23.5), wall thickening in the ascending colon (SUVMax: 26.1), and hypermetabolic areas in the abdomen thought to be due to the spread of the disease in the peritoneum (**Figure 2B**). The systemic treatment of the patient was started with 5-fluorouracil, folinic acid, irinotecan, oxaliplatin (FOLFIRINOX), and bevacizumab while waiting for the MSI results.

**Figure 3:**
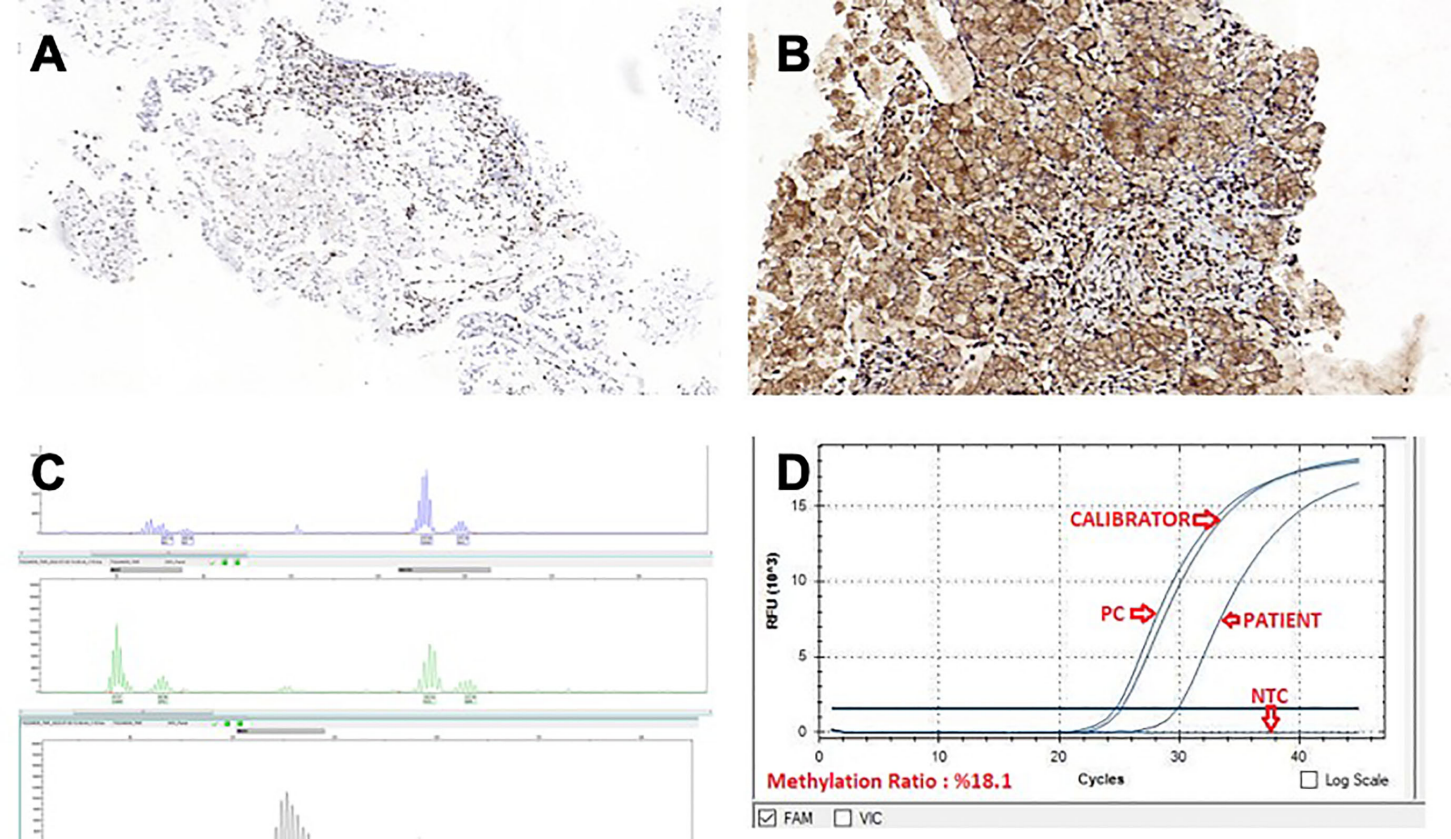
**(A)** MLH-1, IHC x10.0 **(B)** PMS-2, IHC x15.5 **(C)** MSI-H phenotype molecular analysis with PCR **(D)** MLH-1 methylation analysis.

A control CT after 4 cycles of treatment with FOLFIRINOX + bevacizumab regimen showed partial regression, and the treatment was completed to 8 cycles. A PET/CT scan following 8 cycles of FOLFIRINOX + bevacizumab showed a 90% metabolic response (SUVMax: 3). Meanwhile, immunohistochemical evaluation of the MSI panel from the primary tumor showed nuclear dot-like staining patterns for MLH1 (**Figure 3A**) and PMS2 (**Figure 3B**). Molecular analysis with PCR was performed due to suspicious staining patterns for MLH-1 and PMS-2. It revealed MSI-H phenotype (**Figure 3C**). MLH-1 methylation analysis was also performed; hypermethylation in the promoter region of the MLH-1 gene was detected (**Figure 3D**). The systemic treatment was converted to folinic acid, fluorouracil, and oxaliplatin (FOLFOX) combined with pembrolizumab after the status of MSI-H was confirmed. The rationale behind this strategy was the assumption that the primary tumor, metastatic lymph nodes, and peritoneal implants had responded dramatically to triplet chemotherapy + bevacizumab.

**Figure 4:**
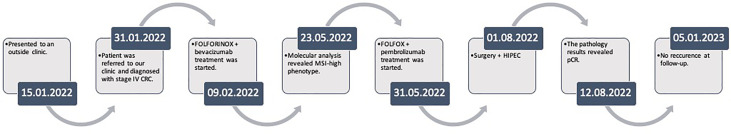
Timeline of the patient’s management.

The patient was treated with additional 4 cycles of FOLFOX concurrently with pembrolizumab with no adverse effects. The MRI prior to surgery showed that the cecal mass regressed and was limited to the mesocolon. The patient underwent a right hemicolectomy, a pelvic and lower abdominal peritonectomy, a total omentectomy, and a diverting loop ileostomy. Hyperthermic intraperitoneal chemotherapy with mitomycin-C was administered for 90 minutes. No major surgical complications occurred, and the stoma was reversed a month after the surgery. The pathology results revealed ypT0N0 (60 lymph nodes). There were no tumor cells in the peritoneal fluid cytology. Pembrolizumab as maintenance treatment was continued after curative resection. The patient was tumor free in his postoperative 6^th^-month follow-up. The treatment was planned to be continued for 24 months if no toxicity or adverse effects were seen. The timeline of the patient is summarized in **Figure 4**.

## Discussion

This case presents a successful treatment of a patient with metastatic MSI mucinous colonic adenocarcinoma with immunotherapy in combination with 5-FU and oxaliplatin based chemotherapy. MSI status is one of the critical factors affecting the treatment approach in mCRC patients (4). While IHC is a more available, cost-effective, rapid, and concordant way to detect MSI status, PCR is a crucial next step to diagnose MSI for inconclusive, borderline cases such as nuclear dot-like staining patterns, as in our patient (8, 9).

Management of mCRC with microsatellite stable status depends on the tumor and patient-related factors, such as performance status and comorbidities, and the aim of the treatment, such as conversion to a resectable state. Usually, a combination of chemotherapy with biological agents like anti-VEGF or anti-EGFR therapy is considered depending on tumor-related factors, including RAS, RAF status, and tumor-sidedness. For MSI-H mCRC patients, pembrolizumab or nivolumab -/+ ipilimumab is recommended as a first-line therapy depending on the results of the KEYNOTE-177 and CM-142 studies (10–12). The Keynote-177 trial revealed 11.1% of complete response and 29.4% of progressive disease in stage IV colorectal patients treated with single-agent pembrolizumab (13). Since the response of BRAF mutant patients to chemotherapy is lower than the wild type, we preferred to start with combination chemotherapy as FOLFIRINOX + bevacizumab until the MSI results were confirmed. Our patient had been a candidate for single-agent pembrolizumab treatment based on the MSI status. However, the MSI tumor’s sensitivity to FOLFIRINOX chemotherapy has been confirmed after 4 months of treatment, and a dramatic response had already been demonstrated in PET-CT even before the commencement of pembrolizumab. Due to the 30% risk of progression with single-agent pembrolizumab in the KEYNOTE-177 trial and our patient’s *in vivo* confirmed sensitivity to chemotherapy, we preferred to continue FOLFOX regimen concurrently with immunotherapy.

Even though chemotherapy and immunotherapy combination is a widely used treatment approach in non-colorectal cancers in both early and advanced settings with satisfactory long-term results, there is no phase III data supporting the use of this combination for mCRC yet (6, 7).

The hypotheses to explain the biological mechanisms underlying resistance to chemotherapy of MSI-associated diseases primarily concern adjuvant fluorouracil-based treatments in non-metastatic CRC. Studies cited in literature often point at the antitumor immune response characterized by the lymphocyte infiltrate of MSI diseases, constituting the basis for the improved prognosis of these patients in early stages. This advantage is supposed to be antagonized by the immunosuppressive effects of chemotherapy that explain the lack of benefit of single agent fluoropyrimidine-based chemotherapy (3). It is important to emphasize that these studies refer to stage II CRCs. According to some authors, adding irinotecan or oxaliplatin counteracts resistance to fluorouracil in MSI tumors (14, 15). However, these hypotheses have not been confirmed in randomized studies. Two phase III randomized trials for resected stage III MSI CRC have been initiated: the ATOMIC study, which is evaluating FOLFOX (5-FU/LV + oxaliplatin) ± atezolizumab for 6 months plus maintenance with atezolizumab or placebo for 6 months (NCT02912559), and the POLEM study (NCT03827044), which aimed to evaluate the efficacy of 24 weeks of FP versus 12 weeks of FP plus oxaliplatin ± avelumab for MSI or patients with POLE mutation. However, the POLEM study has been terminated due to challenges in patient recruitment.

A phase Ib trial evaluating the effect of chemoimmunotherapy, pembrolizumab, in combination with a modified FOLFOX regimen in metastatic colorectal patients has been completed (16). In this trial, 6.7% of the patients had a complete response, and patients with advanced disease at the time of diagnosis had reduced tumor burden and became eligible for definitive surgery. 6.7% of the patients had grade 3 or 4 toxicity; the remaining patients tolerated the treatment well. Similar to our case, Copur et al. (17) published a case of a locally advanced colon cancer treated with FOLFOX + pembrolizumab, resulting in a pathologically complete response.

BRAF mutation is seen in 38.9% of the patients with MSI, while only 9.3% of patients with MSS CRC have a BRAF mutation, and 20.4% of the patients with BRAF mutation were MSI (8). In the subgroup analysis of KEYNOTE-177 study, BRAF wild type patients seem to have better survival with immunotherapy compared to chemotherapy (for OS; HR 0.72 vs. 0.55 for BRAF mutant and wild type, respectively).

BRAF mutation is associated with poor prognosis reducing the benefits derived from MSI even in the earlier disease setting. The BRAFV600E mutation has been a poor prognostic factor in both MSI and MSS patients underlining the importance of these biomarkers for the management of patients at recurrence (18). Thus, chemoimmunotherapy may be explicitly considered for patients with BRAF mutant and MSI-H tumors since it’s a promising approach with a tolerable toxicity.

While being a case report, the major limitation to propose chemotherapy in combination with immunotherapy as a novel treatment for MSI mCRC with in general, this index case can be didactic to plan future studies of this promising approach.

## Data availability statement

The original contributions presented in the study are included in the article/supplementary material. Further inquiries can be directed to the corresponding author.

## Ethics statement

Written informed consent was obtained from the individual(s) for the publication of any potentially identifiable images or data included in this article. Written informed consent was obtained from the participant/patient(s) for the publication of this case report.

## Author contributions

Writing original draft: AM, EA, LO. Supervision: LO, EA, SE. All authors participated in the revision of the manuscript. All authors contributed to the article and approved the submitted version.
